# Emotional Intelligence as Evaluative Activity: Theory, Findings, and Future Directions

**DOI:** 10.3390/jintelligence11060125

**Published:** 2023-06-20

**Authors:** Michael D. Robinson, Muhammad R. Asad, Roberta L. Irvin

**Affiliations:** Department of Psychology, North Dakota State University, Dept. 2765, P.O. Box 6050, Fargo, ND 58108-6050, USA

**Keywords:** emotional intelligence, ability, evaluation, bipolarity, extremity

## Abstract

The question of whether ability-related emotional intelligence (ability EI) predicts important life outcomes has attracted considerably more attention than the question of what ability EI consists of. In the present paper, the authors draw from the attitude and emotion literatures to suggest that the evaluation dimension of meaning is likely key in understanding how ability EI operates. Measures of ability EI predict the extent to which individuals can accurately evaluate words and measures of the latter type act as emotional intelligence measures. Extending this analysis, the paper reviews recent sources of data linking ability EI to attitudinal processes, such as those involved in attitude–behavior relationships and affective bipolarity. Individuals with high EI appear to experience their affect in more bipolar terms and they display evidence of greater decisiveness in their evaluations. Pursuing links of the present type will allow researchers to generate new predictions concerning the ability EI construct.

## 1. Introduction

Relative to the intelligence literature, the emotional intelligence literature has a shorter history. The construct was formally proposed in 1990, popularized in 1995, and redefined in 1997, as reviewed by Barchard et al. (2016). Emotional intelligence (EI) can be assessed in trait-related terms (i.e., as a set of personality traits) or it can be assessed through the use of ability-related tests such as the MSCEIT (Mayer et al. 2003), the STEU and the STEM (MacCann and Roberts 2008), or the NEAT (Krishnakumar et al. 2016). Trait-related and ability-related tests of EI do not correlate very highly with each other (Brackett et al. 2006; Joseph and Newman 2010) and the focus of the present paper primarily concerns ability-related conceptions (Mayer et al. 2008).

In addition to psychometric considerations, researchers have asked questions such as whether individual differences in ability-related emotional intelligence (ability EI) matter with respect to important life outcomes. Several papers link ability EI to social functioning, though relevant findings seem to depend on one branch of EI (management) and do not appear to be fully consistent across studies (Lopes et al. 2004; Lopes et al. 2011). There is some link between ability EI and outcomes such as health (Martins et al. 2010) and well-being (Sánchez-Álvarez et al. 2016), though these relationships are modest (in the .20 range) and often do not replicate (e.g., Di Fabio and Kenny 2016). Ability EI has been linked to better academic performance, though the magnitude of this relationship is modest and may be particularly evident in humanities courses (MacCann et al. 2020b). Links between ability EI and work-related behaviors or performance are often not significant when controlling for personality and cognitive ability (Miao et al. 2017; O’Boyle et al. 2011). These findings have been characterized as disappointing by some commentators (Matthews et al. 2012; Ybarra et al. 2014) and even advocates of ability EI have suggested that relationships between ability EI and behaviors or performance could be complicated (Joseph and Newman 2010; Mayer et al. 2016).

One important development in intelligence research occurred when researchers began focusing on how variations in intelligence operate—namely, how they manifest themselves in stimulus processing and reactivity (Jensen 2006). Given the state of the ability EI literature, similar directions can be advocated (Fiori 2009; Miners et al. 2018). Several lines of research hint at what this next generation of ability EI research might look like. MacCann et al. (2011) found that participants with higher levels of ability EI engaged in problem-focused coping to a greater extent and this difference in coping strategies explained a portion of the link between ability EI and grade point averages. Megías-Robles et al. (2019) found that individual differences in ability EI mattered for emotion regulation strategies, with participants with high EI more likely to engage in reappraisal and participants with low EI more likely to engage in suppression. A line of research indicates that ability-related variations in EI, but not trait EI, facilitates cognitive control within tasks that involve emotional processing (Checa and Fernández-Berrocal 2019; Gutiérrez-Cobo et al. 2017).

In the current paper, we do not focus on coping, emotion regulation, or cognitive control per se. Rather, we focus on evaluative activity, which, we think, is core to how ability-related variations in emotional intelligence operate. Individuals with high EI, more or less, may be experts in evaluating the stimuli and conditions that they encounter (Hoemann et al. 2021). We begin by highlighting the centrality of evaluations to a variety of phenomena, such as connotative meaning, attitudes, and emotions. We then argue that individual differences in emotional intelligence can be conceptualized in terms of individual differences in the likelihood, precision, and certainty with which evaluations are made. After presenting several sources of data that have been amassed in support of this perspective, we outline future directions of research that can build on the present analysis.

## 2. Evaluation’s Centrality to Affective and Emotional Meaning

Emotional intelligence can be defined in terms of abilities related to the identification, expression, understanding, management, and use of emotions (Kotsou et al. 2019). Although definitions such as these emphasize real-world emotional phenomena, assessments of ability EI concentrate on varieties of emotion-related knowledge, typically in the absence of emotional experiences. Much of this knowledge is likely to be evaluative in nature. For example, one must evaluate the situations of the STEU (MacCann and Roberts 2008) or the NEAT (Krishnakumar et al. 2016) to make accurate inferences concerning the emotions that characters would experience. Evaluative meaning is core to many of the answer options that test-takers are presented with, which often consist of emotion words (e.g., sad, grateful, angry). When simpler stimuli such as landscapes or art images are presented (Mayer et al. 2003), these stimuli must also be evaluated in order for the test-taker to provide accurate answers. Participants who are more inclined to evaluate the objects that they are exposed to may typically receive higher scores on ability EI tests, provided that their evaluations accord with other test-takers (or experts) to any extent. Ability EI tests certainly assess things beyond evaluative knowledge, but the possession and use of such knowledge may be key. We provide empirical support for this idea in the next section.

One reason for focusing on evaluations, as this review does, is that they are key to any type of meaning that is personal and subjective rather than impersonal and descriptive (Epstein 2003). This point was repeatedly made by Osgood and colleagues, who asked participants to characterize numerous concepts and stimuli in terms of semantic contrasts such as good–bad, weak–strong, hard–soft, and so on (Osgood 1962). Factor analyses of these ratings reveal that the connotative space of meaning is (universally) anchored by three dimensions termed evaluation (is the stimulus good, bad, or in between?); potency (is the stimulus strong, weak, or in between?); and activity (is the stimulus active, passive, or in between?). Evaluation is the first factor of this space, meaning that the most robust distinctions are made with respect to the evaluative dimension of meaning relative to the other two dimensions (Osgood et al. 1957). If emotionally intelligent people are skilled at assigning affective meaning to the stimuli and situations they encounter, as we have suggested is the case, they should be skilled at evaluation, which is the most central component of the affective meaning system (Osgood 1962).

Evaluations are certainly central to emotion. All appraisal theories of emotion posit that appraisals related to evaluation (e.g., is this situation pleasant or unpleasant?) shape emotional reactions (Roseman and Smith 2001) and the “pleasantness check” is thought to occur early in the emotion generation process (Scherer 2009). All emotions can be characterized in terms of their valence—that is, whether the emotion feels pleasant or unpleasant (Barrett and Russell 1999)—and this dimension of emotion is thought to organize the diverse emotional experiences that we have (Russell 2009). Indeed, all unpleasant emotions tend to covary with each other, as do all pleasant emotions, highlighting the centrality of valence (or evaluation) to the emotion space (Watson 2000). Barrett (2006) summarizes this literature by proposing that valence is the basic building block to all emotional phenomena.

Our analysis will emphasize the close affinity that exists between the attitude and emotion literatures (Cacioppo et al. 1997), given that attitudes are, at their core, evaluations of stimuli, whether concrete objects or values or ideas (Eagly and Chaiken 1993). Attitudes are thought to structure the environment, meaning that a person without strong or retrievable attitudes would experience difficulties knowing how to relate to what they encounter (Lewin 1935). In a compelling line of research supporting this point, Fazio and colleagues show that accessible (easy to retrieve) attitudes guide attention (Roskos-Ewoldsen and Fazio 1992), ease decision-making (Fazio et al. 1992), reduce stress (Fazio and Powell 1997), and render it more likely that the person acts in a manner consistent with their attitudes (Fazio and Williams 1986). In studies reported below, we leverage the attitude literature to make the case that individuals with higher levels of ability EI are, in a sense, expert evaluators (Hoemann et al. 2021).

One difference between the ability EI and attitude domains is that many of the attitudes that we have (e.g., a liking for mint chip ice cream) would not seem to lend themselves to an ability-related analysis. However, we believe that abilities are involved in the attitude domain nonetheless. For example, in deciding how we feel about a stimulus, we must retrieve stimulus-relevant previous experiences, many of which could extend well into the personal past (Cunningham and Zelazo 2007). Further, such previous experiences could be numerous and would, therefore, need to be integrated in a skillful manner (Rolls and Grabenhorst 2008). The possession of most attitudes requires experience with the domain and experts make evaluations in a manner that is different (e.g., more integrative) than novices (Brauer et al. 2004). Further, attitudinal judgments can be practiced and doing so increases the accessibility of the relevant attitudes as well as their ability to guide behavior (Fazio 2007). Finally, we suggest that attitudes can be incorrect when they fail to adequately capture the self’s experiences, either past or future. In this connection, Wilson and colleagues show that some attitudes, despite guiding behavior in the present, are demonstrably incorrect (e.g., Wilson et al. 1993). The attitude domain is, therefore, one that involves skills.

Although the results that we report are novel, there is precedent for the idea that some people, more so than others, are either more inclined toward, or more skilled at, making evaluations. As an example, individual differences in mindfulness are thought to sensitize individuals to the affective features of their existence, in turn facilitating capacities-related emotion regulation and self-regulation (Teper et al. 2013). Of greater pertinence, Jarvis and Petty (1996) hypothesized that individuals differ in their need to evaluate—that is, their tendencies to care about and evaluate the stimuli and issues that they are exposed to. Jarvis and Petty (1996) found support for variations along this individual difference continuum and showed that participants with a higher need to evaluate had more extreme attitudes. They were also more likely to spontaneously engage in evaluative activity (e.g., with respect to unfamiliar paintings).

Barrett (2006) proposed a seemingly parallel set of individual differences termed valence focus. Valence-focused individuals emphasize the valence (pleasant–unpleasant) dimension in their self-reports of emotional experience and exhibit tendencies—such as in perceiving emotional stimuli (Barrett and Niedenthal 2004) or reacting to the events of the day (Pietromonaco and Barrett 2009)—that implicate greater sensitivity to evaluative components of meaning. Although ability-related variations in EI should probably not be equated with need to evaluate or valence focus (particularly given that the personality-related correlates of valence focus appear to be different: Barrett 2006), the operations involved in ability EI could function similarly (also see Fiori and Ortony 2021). We build this case in a systematic manner, highlighting older and newer lines of research from our laboratory.

## 3. Results Involving a Word Evaluation Task

In the creation of new ability EI tests, investigators often embrace new technologies such as multimedia assessments (Roberts et al. 2010). However, if evaluation is core to emotional phenomena (Barrett 2006), we might think about developing deliberately simple or basic measures, such as those involving word evaluations. Moeller et al. (2012) developed such a test, which simply asked participants to evaluate (1 = negative; 8 = positive) 100 common, well-known words (e.g., world, passion, illness, gossip) that varied in valence norms (Bradley and Lang 1999; Meier and Robinson 2004). Positive words were evaluated more favorably than negative words, partial η2 = .89, but we were interested in subtler distinctions involving the stimuli. For each participant separately, for example, we correlated the evaluations that a participant made with mean evaluations from the whole sample, with higher correlations reflective of greater evaluative insight (Legree et al. 2005).

That word evaluation tests of this type relate to emotional intelligence was examined in study 4 of Krishnakumar et al. (2016), who administered the North Dakota emotional abilities test (NEAT), which uses the situational judgment test format (Libbrecht and Lievens 2012) to assess individual differences in emotion perception (inferring the emotions that would be experienced by characters in emotional situations), emotion understanding (knowing which emotions would tend to co-occur or transition from one to the other), and emotion management (endorsing ways of responding to emotional situations that are deemed more effective by an expert sample). In this particular study, the word evaluation task was scored in terms of discrepancies from evaluation means, with higher scores indicative of poorer (or less normative) evaluations (Legree et al. 2005). The correlation between the NEAT and word evaluation accuracy scores was r = −.49, indicating that there is substantial overlap between evaluative expertise and ability-related emotional intelligence. For individual branches, these correlations were −.40, −.42, and −.45 for emotion perception, understanding, and management, respectively, indicating that all branches of the ability EI test seem to benefit from simple evaluation skills.

Furthermore, the word evaluation test acts as an emotional intelligence test. Moeller et al. (2012) pursued the premise that interpersonally cold individuals, who are hostile, disengaged, or quarrelsome (Horowitz et al. 2006), may lack the emotional repertoire that allows them to bond with others (Moskowitz 1994, 2005), which should be evident in terms of lower levels of ability EI. This premise was supported in studies that used an emotion perception task involving faces (study 1), dynamic video stimuli (study 2), an emotional understanding task (study 3), and with respect to the normative accuracy of word evaluations (study 4). In the latter case, for example, the correlation between participant word evaluations and evaluation norms was .36 at low (−1 SD) levels of interpersonal coldness and .27 at high (+1 SD) levels, ηp2 = .07. More generally, the fact that results were parallel across tasks and studies supports the idea that what is assessed by word evaluation accuracy shares some affinity with what is assessed by more complex EI tests, including one (the STEU: MacCann and Roberts 2008) that has been extensively validated (Libbrecht and Lievens 2012).

**Figure 1:**
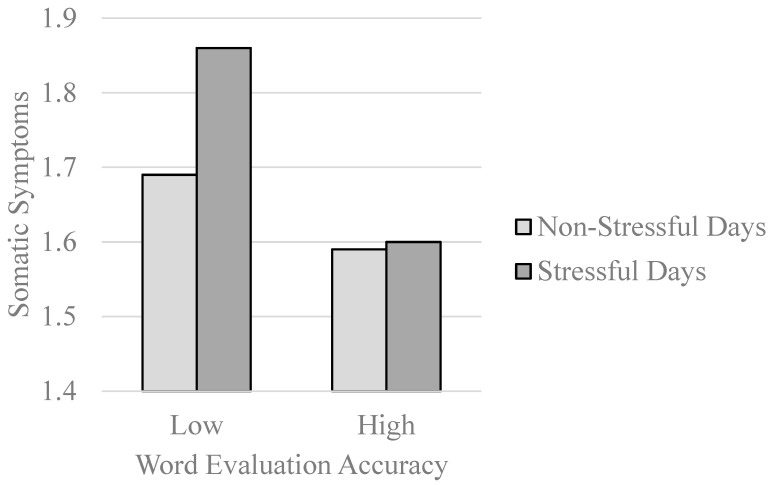
Word evaluation accuracy as a moderator of relations between daily stressors and somatic symptom experiences (re-graphed results from Robinson et al. 2012).

Robinson et al. (2012) then used a word evaluation task to examine regulatory processes in daily life. Borrowing from cybernetic frameworks for self-regulation (Carver and Scheier 1998) and emotion regulation (Robinson et al. 2010), Robinson et al. (2012) reasoned that a better attuned affective monitor (Teper et al. 2013) would allow individuals to better regulate their responses to daily stressors (Compton et al. 2008). In study 2 of Robinson et al. (2012), participants completed a daily diary protocol (Bolger et al. 2003) for 15 consecutive days. On each day, participants indicated how many stressful events had occurred to them and they also reported on their daily experiences of somatic symptoms (e.g., headaches, nausea), which are often exacerbated by stressors (Pennebaker 1982). As displayed in Figure 1, stressors precipitated somatic complaints at low levels of evaluative accuracy, but not at high levels. The skills assessed by a simple word evaluation task, therefore, covary with other skills that are beneficial in regulating daily stressor reactivity.

**Figure 2:**
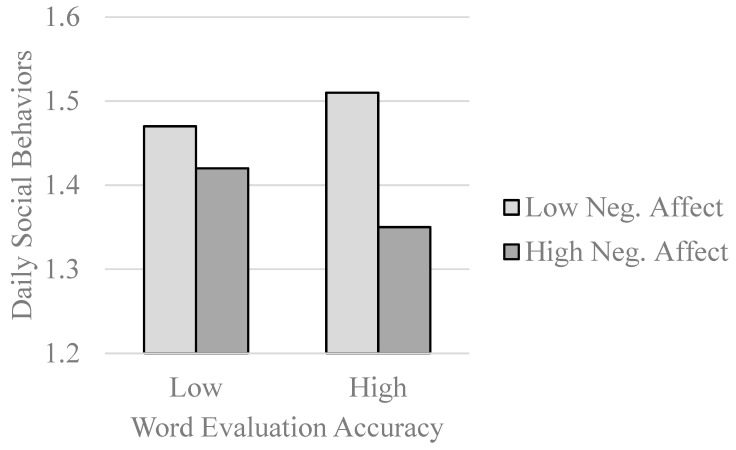
Word evaluation accuracy as a moderator of relations between daily negative affect and daily social behavior (re-graphed results from Moeller et al. 2014).

In the domain of self-regulation, being attuned to affective signals could permit one to make wise decisions in response to current affective states (Clore et al. 2001). Negative affective states are thought to encourage withdrawal (Watson 2000), in part because interacting with others when one is feeling upset can result in interpersonal conflict (Forgas 2002). In three studies, Moeller et al. (2014) examined whether affectively attuned individuals (i.e., those with higher EI levels) would modulate their social behavior in response to naturally occurring variations in negative affect. On days on which people were experiencing higher levels of negative affect, participants engaged in fewer social behaviors, but this relationship was stronger among participants who were more skilled in the word evaluation task (see Figure 2). Results were parallel across other ability EI measures and the convergence of findings across studies again attests to the point that a word evaluation measure acts as an emotional intelligence measure.

In summary, we created a simple word evaluation measure (that exhibits modest correlations with cognitive ability: *r* = .18) to assess basic skills related to evaluation. Consistent with the idea that basic evaluation skills are linked to ability EI, Krishnakumar et al. (2016) found that individuals whose word evaluations better matched norms also achieved higher levels of ability EI and relationships of this type were evident across all branches of the ability EI test. Three other investigations support the point that the word evaluation test acts as an emotional intelligence test. For example, individuals who are better able to evaluate words are better able to regulate their responses to daily life stressors (Robinson et al. 2012). These results support the idea that individuals with high EI, relative to individuals with low EI, appear to be more skilled in making evaluations.

## 4. Bipolarity in Affect and Evaluation

Emotion scholars have long been interested in the structure of affective experience (e.g., Schlosberg 1941). Of particular interest is the question of whether pleasant (positive) and unpleasant (negative) emotional experiences are bipolar to each other (e.g., if one is experiencing high levels of positive affect, one is necessarily experiencing low levels of negative affect) or can vary independently, such that one could experience at least moderately intense positive and negative affects at the same time (Russell 2017). Some scholars, such as Wundt, Schlosberg, and Russell (as reviewed in Barrett and Russell 1999), have favored bipolarity (strong inverse correlation) and others, such as Bradburn, Tellegen, and Watson (as reviewed in Reich et al. 2003), have favored independence.

There have been some attempts to reconcile bipolarity and independence. For example, two investigations conclude that a bipolar (pleasant–unpleasant) factor might exist in addition to relatively independent positive and negative affect factors (Leue and Beauducel 2011; Tellegen et al. 1999). However, the more interesting suggestion, from our point of view, is the idea of individual differences, with some people exhibiting a greater degree of bipolarity in their affective experiences than others (Rafaeli et al. 2007). In support of the reality of such individual differences, Dejonckheere et al. (2018) found that within-subject correlations between positive and negative affect (with reporting occasion as the unit of analysis) varied from −.82 (strong bipolarity) to .12 (independence).

There are differences of opinion, however, concerning whether higher levels of bipolarity are beneficial or problematic. Related to the former possibility, some scholars suggest that sophisticated affect systems tend toward independence, such that it would be hazardous to infer negativity levels from positivity levels (Ong et al. 2017; Reich et al. 2003). For example, it is thought that older individuals (Hay and Diehl 2011) or those from cultures with a history of dialectical thinking (Hui et al. 2009) may, at least under some circumstances, experience moderately high levels of both positive and negative affect at the same time (Ong et al. 2017). These ideas have not produced a very coherent body of findings (Grossmann et al. 2016; Hay and Diehl 2011), however, and one can amass multiple arguments for why greater bipolarity (lesser independence of positivity and negativity) would be more functional.

In the attitude literature, a mix of positive and negative evaluations appears to be problematic. Mixed evaluations are experienced aversively (van Harreveld et al. 2015) and ambivalent attitudes are also less predictive of behavior as well as being less stable over time (Conner and Armitage 2008). To function effectively, one’s attitudes may need to possess reasonably high degrees of bipolarity—that is, liked objects should not be disliked and disliked objects should not be liked (Fazio 2000; Fazio and Powell 1997). In fact, mixed affective states often generate approach–avoidance conflict, which is pernicious for multiple reasons (Aupperle et al. 2011; Miller 1944; Robinson et al. 2008). In the emotion realm as well, bipolarity may provide better guidance concerning the current conditions of the self-environment interface (Russell 2017).

We have suggested that individuals with high EI are more capable evaluators. Given that evaluation is a bipolar dimension (Osgood 1962), the affective states of individuals with high EI may also tend toward greater bipolarity. In the studies that follow, ability EI was assessed using the NEAT (Krishnakumar et al. 2016), which has performed well in many studies (e.g., Krishnakumar et al. 2017, 2019a, 2019b; Robinson et al. forthcoming b; Robinson et al. 2019) and which, despite its branches, primarily assesses EI in global terms (Krishnakumar et al. 2016).

Robinson et al. (2020) conducted three studies that assessed experiences of positive and negative affect at work, during the previous month (studies 1 and 2), or in daily life (study 3). In all cases, the NEAT moderated relations between positive and negative affect, which were more bipolar among individuals with higher ability EI scores. In study 2, for example, a median split on the ability EI measure revealed that experiences of positive and negative affect were more bipolar above the median (−.49) than below it (−.17). These results, which were replicated using continuous predictors in moderated multiple regression, suggest that the emotional experiences of individuals with high EI tend toward greater clarity; that is, if such individuals are experiencing higher levels of positive affect, they are experiencing lower levels of negative affect, and vice versa. By contrast, individuals with low EI may be more confused about how they feel, at least from an affect structure perspective (Russell 2017).

In a more recent paper (Robinson et al. 2023), we considerably extended this analysis. In study 1, participants completed the NEAT as well as a number of tasks suited to examine within-person bipolarity. In an attitude task, participants were asked to indicate how positively (1 = not positive at all; 7 = very positive) and negatively (1 = not negative at all; 7 = very negative) they felt about a series of 20 attitude objects (e.g., chemicals, exercising, mornings, secrets), presented in a randomized order. For each participant separately, we then calculated a bipolarity coefficient by correlating positivity ratings with negativity ratings (n = 20; M across participants = −.85). Higher levels of ability EI were linked to greater bipolarity, β = −.38. Of additional importance, ability EI predicted how variable evaluative ratings were (across the 20 objects) and this was true for both positive, r = .37, and negative, r = .40, ratings. The EI–bipolarity relationship remained significant when controlling for evaluation variability, however, indicating that variability, per se, could not account for the bipolarity relationship that was observed.

In another task, we asked participants to report on their positive (e.g., happy, excited) and negative (e.g., angry, fearful) reactions to a series of 10 emotional images (Lang et al. 2005). For each participant separately, we then calculated a bipolarity coefficient in a manner parallel to that described above (n = 10; M across participants = −.58). Participants with higher EI levels displayed greater bipolarity, β = −.23, and they also exhibited greater variability (across stimuli) in their positive, r = .22, and negative, r = .33, emotional reactions. With respect to this task, the EI–bipolarity relationship was reduced to non-significance when controlling for the variability of emotional responses. Regardless, that individuals with high EI display more variable emotional reactions is part of the point, in that EI should be associated with patterns of emotional responding that are more situation- or stimulus-specific (Waugh et al. 2011).

In study 2 of Robinson et al. (2023), we applied a bipolarity analysis to experiences of job satisfaction, which predict numerous organizational outcomes such as attendance, turnover intentions, job performance, and workplace civility (Judge and Klinger 2008). Most scales of job satisfaction contain both positively (e.g., “I find real enjoyment in my work”) and negatively (e.g., “I consider my job rather unpleasant”) keyed items, the latter of which are typically reverse-scored. Rather than reverse-scoring the latter items, we computed two scores within three employee samples—a job satisfaction score, capturing favorable attitudes toward one’s work, and a job dissatisfaction score, capturing unfavorable attitudes. Bipolarity with respect to this important life domain would result in a stronger inverse correlation between job satisfaction (favorable attitudes) and job dissatisfaction (unfavorable attitudes).

**Figure 3:**
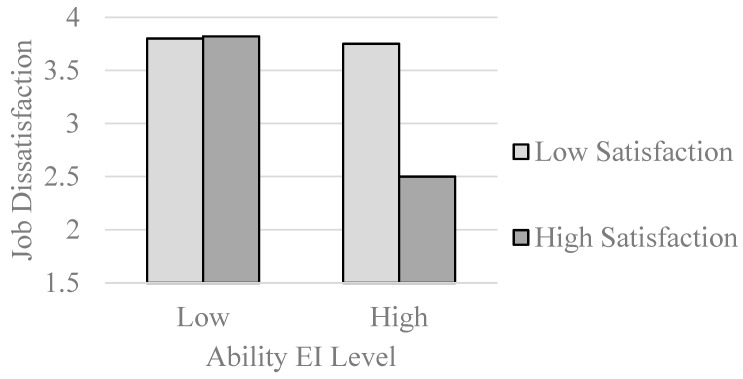

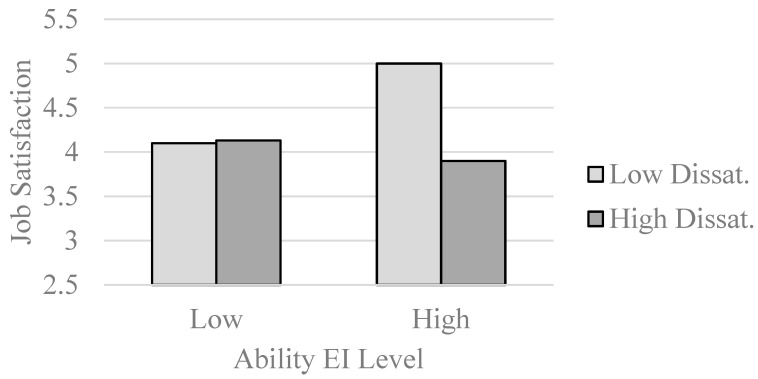
Ability emotional intelligence moderates the relationship between job satisfaction and job dissatisfaction (**top panel**) and job dissatisfaction and job satisfaction (**bottom panel**).

Median splits on the NEAT variable produced descriptive statistics consistent with expectation. Below the median (low EI) satisfaction–dissatisfaction correlations were −.48 (sample 1), −.03 (sample 2), and −.34 (sample 3). Above the median (high EI), these correlations were −.64, −.63, and −.73. Multiple regression results, which examined whether continuous variations in ability EI moderated the relationship between satisfaction and dissatisfaction or between dissatisfaction and satisfaction, consistently resulted in interactions and estimated means for sample 2 are displayed in Figure 3. As can be seen in the figure, both satisfaction–dissatisfaction and dissatisfaction–satisfaction relationships were more inverse at higher levels of ability EI. That is, if employees with high EI are satisfied with their jobs, they are not dissatisfied (and vice versa). Employees with low EI, by contrast, are more prone to mixed evaluations of their jobs.

## 5. Further Insights Based on an Attitudinal Analysis

We suggest that individuals with high EI are experts in evaluation and evaluation is the central feature of the attitude construct (Eagly and Chaiken 1993). As this is true, new insights concerning ability EI can be achieved by integrating these literatures to a greater extent. The concept of attitude strength (Petty and Krosnick 1995) merits particular attention because researchers demonstrate, in many different ways, that some attitudes are stronger than others. Strong attitudes (relative to weak ones) are more stable over time and more predictive of perception and behavior (Luttrell and Sawicki 2020). Predictors of attitude strength are numerous (Krosnick and Smith 1994) and we primarily focus on certainty (how certain a person feels concerning an attitude report), extremity (how polarized from the midpoint the attitude report is), and cognitive–affective consistency (whether cognitive and affective responses to the attitude object are consistent or inconsistent).

In a series of three studies, Irvin et al. (2023) conducted research of this type, again assessing variations in ability EI in terms of total NEAT scores (Krishnakumar et al. 2016). Study 1 asked individuals to make several judgments concerning a series of single word attitude objects (e.g., capitalism, dentists, gossip, politics, romance, science, etc.). Following the tripartite distinction between affect, behavior, and cognition (ABC: Breckler 1984), participants were asked how positive their thoughts concerning each attitude object were (1 = negative; 4 = neutral; 7 = positive), whether each attitude object made them feel happy or unhappy (1 = unhappy; 4 = neutral; 7 = happy), and whether they would approach or avoid the attitude object (1 = definitely avoid; 4 = neutral; 7 = definitely approach). Participants were also asked how certain they were concerning their attitudinal responses (1 = not certain; 7 = very certain), again concerning each object.

Higher levels of EI were predictive of greater (average levels of) attitudinal certainty, β = .41. In addition, we calculated extremity scores for the affect, behavior, and cognition ratings by calculating distance from the midpoint (e.g., a response of 4 would be scored as 0). Participants with higher levels of EI had more extreme attitudes, whether defined cognitively, β = .37, affectively, β = .31, or behaviorally, β = .41. The behavioral response is particularly notable because the findings suggest that individuals with high EI are likely to exhibit more pronounced approach or avoidance behavior, depending on their evaluations of the attitude object (Lewin 1935). In addition, we calculated cognitive–affective, cognitive–behavioral, and affective–behavioral consistency scores by correlating these measures with each other, with attitude object (n = 20) as the unit of analysis. Participants with higher EI tended toward greater consistency in their thoughts, feelings, and behaviors concerning particular objects, as EI was a significant predictor of cognitive–affective consistency, β = .22, cognitive–behavioral consistency, β = .27, and affective–behavioral consistency, β = .23.

In study 2 of Irvin et al. (2023), we examined the stability criterion of attitude strength (Luttrell and Sawicki 2020) by asking for affective and behavioral ratings of objects early on in the session and then by re-presenting the objects and questions later in the session, this time in a different randomized order. Of note, participants were told that this was not a memory task; rather, we wanted fresh evaluations each time. Even so, and for each participant separately, we correlated time 1 ratings with time 2 ratings, which resulted in two stability coefficients, one for affective responses and one for behavioral responses. Ability-related variations in EI predicted the stability of both affective, β = .27, and behavioral, β = .35, responses across time. That is, the attitudinal responses of participants with high EI appeared to be stronger according to the stability criterion.

An additional purpose of study 2 was to examine the scope and range of the extremity effect identified in study 1. Participants were asked to evaluate abstract paintings that they almost certainly had not seen before (1 = I do not like this painting at all; 7 = I like this painting very much). They were also asked to evaluate metaphors (e.g., “creativity is a toaster”, “a bird is nature’s airplane”) that ranged from very good to very poor, using the norms of Katz et al. (1988). Participants rated how good, apt, or pleasing each metaphor was (1 = not good, apt, or pleasing; 7 = very good, apt, or pleasing). Finally, participants guessed how pleasant or unpleasant (1 = very unpleasant; 7 = very pleasant) the objects signified by obscure foreign languages (e.g., kaamos, prosim) were. For each of these three tasks, we computed extremity scores (distance from the neutral midpoint) and then averaged across objects. Participants with higher EI levels tended to make more extreme evaluations of all three classes of objects, whether paintings, β = .20, metaphors, β = .29, or foreign language words/objects, β = .18. Although the magnitudes are not large, the consistency of the results is impressive, and it appears that individuals with high EI evaluate many objects more definitively.

In study 3, we (Irvin et al. 2023) applied the extremity analysis to two very important objects—the self and one’s job. With respect to the self, participants were asked whether personality statements (e.g., I don’t talk a lot, I have a vivid imagination) accurately described them (e.g., 1 = very inaccurate; 3 = neither inaccurate nor accurate; 5 = very accurate). The statements were mixed in the sense that items referred to multiple traits and both positively keyed and negatively keyed items were present. Rather than scoring personality traits, though, we simply scored each rating in terms of its deviation from the neutral midpoint. Participants with higher EI levels report higher levels of agreement or disagreement with the statements and this is true in four samples (sample 1: β = .17; sample 2: β = .35; sample 3: β = .37; sample 4: β = .30). Ability EI, therefore, seems to facilitate greater certainty concerning the self’s attributes.

The same four samples (of employees) also completed job satisfaction scales. All scales were bipolar in nature (e.g., 1 = very dissatisfied; 3 = neutral; 5 = very satisfied) and we could, therefore, score all answers in terms of deviations from a neutral midpoint. In all cases, higher levels of ability EI are linked to greater extremity in these ratings (sample 1: β = .26; sample 2: β = .27; sample 3: β = .36; sample 4: β = .32). That is, employees with higher EI levels are more certain of whether they like their jobs or not. These data attest to the importance of the phenomena identified by Irvin et al. (2023). For example, on the basis of the job satisfaction results, we would expect employees with high EI to be more committed to their jobs when they like them and less committed to them when they do not.

## 6. Implications, Analysis, and Future Directions

Evaluative activity transforms a meaningless environment into one that possesses meaning for the individual (Osgood 1962). As emotions are generated on the basis of evaluative activity (Scherer 2009) and because evaluation is core to emotions themselves (Barrett 2006), it makes sense to posit that individuals who obtain higher emotional intelligence scores (on ability tests) are, among other things, experts at evaluation. That is, they may be more prone to evaluate objects and experiences and they may be more certain, or decisive, concerning the evaluations that they make. In the present paper, we pursued such ideas in both theoretical and empirical terms. Theoretically, for example, we suggest that considerable progress could be made by linking the emotional intelligence literature to that concerned with attitude-related processes.

That emotionally intelligent individuals are experts at evaluation is supported by a fairly substantial correlation between emotional intelligence levels, assessed in standard terms (Krishnakumar et al. 2016), and performance in a word evaluation task. Specifically, participants with higher EI made evaluations of words that agreed with word evaluation norms to a greater extent. It is also shown that word evaluation accuracy acts as an emotional intelligence test. Among other relevant findings, Moeller et al. (2014) showed that participants obtaining higher word evaluation accuracy scores were more likely to alter their behavior on the basis of their daily emotional experiences. Although we would probably not recommend the word evaluation measure over other measures of ability EI, it is, nonetheless, an interesting test that arguably captures at least one core of the sorts of abilities that ability EI tests measure.

Facility with the evaluation dimension would reasonably result in higher levels of bipolarity, given that evaluation itself is a bipolar dimension (Eagly and Chaiken 1993). Consistent with this idea, Robinson et al. (2020) found that participants with higher levels of EI exhibited greater bipolarity in their positive and negative affective states and in daily life. A more general case for links between EI and bipolarity was made by Robinson et al. (2023). In their evaluations of words and in their simulated and actual emotional responses to stimuli, participant with higher EI displayed more inverse within-person relations between their positive and negative evaluations and/or their levels of positive and negative affect. The importance of such dynamics was highlighted in an analysis of experiences of job satisfaction and job dissatisfaction, which were more inverse (i.e., bipolar) at higher levels of EI. It therefore appears that individuals with high EI are more certain as to whether they like or dislike their jobs.

This evaluation-related perspective on ability EI was reinforced in a second series of studies by Irvin et al. (2023). Participants with high EI, relative to low EI, were more certain about their attitudes and their evaluative ratings were more polarized. For example, high EI participants made evaluations of abstract paintings that were more extreme, either liking or disliking them to a greater extent. The real-world manifestations of these tendencies were pursued in a number of work employee samples. Participants with high EI were more certain as to whether particular personality statements described them or not (as defined by greater agreement or disagreement with the items) and their ratings of job satisfaction were also more polarized away from the job satisfaction midpoint (neutrality). It appears that emotionally intelligent individuals are more decisive with respect to the evaluations that they make, likely in part because they engage in evaluative processing more frequently.

EI researchers have spent the vast majority of their time developing tests and/or investigating whether such tests predict distal outcomes such as well-being or job performance. As pointed out by Fiori and Ortony (2021), a much smaller body of research has sought to understand what ability EI is linked to from a process-oriented perspective (also see Gutiérrez-Cobo et al. 2016). By better understanding the processes involved in ability EI, we may better understand what these individual differences should predict. In agreement with Ybarra et al. (2014), we think that the processes involved in EI are dynamic rather than static in nature. For example, they appear to be linked to more positive evaluations of some stimuli, but more negative evaluations of other stimuli, depending on the nature of the stimuli involved. What these tests should predict, therefore, depends on current stimulus conditions or situations that are encountered.

We suggest that the processes involved in ability EI overlap with the processes ascribed to need to evaluate (Bizer et al. 2004; Jarvis and Petty 1996) or valence focus (Barrett 2006; Barrett and Niedenthal 2004), but these models are not perfect because they do not speak to abilities (nonetheless, it would be informative to know whether individuals with high ability EI would score higher in need to evaluate or valence focus). From an ability-related standpoint, EI seems to overlap with descriptions of emotional flexibility (Beshai et al. 2018) or psychological flexibility (Kashdan and Rottenberg 2010). These models suggest that psychological health can be defined, in part, in terms of situation-appropriate responding. For example, Waugh et al. (2011) found that resilient individuals, relative to non-resilient individuals, exhibited more pronounced emotional reactivity to both positive and negative stimuli. That is, their emotion systems were attuned to the stimuli that they encountered. It is very likely that emotionally intelligent individuals would exhibit the same pattern (Fiori and Ortony 2021). If so, our understanding of ability EI would be enhanced by a focus on the processes and attributes suggested by these models. Among other applications of these models, we might expect participants with high EI to be more comfortable with their emotions (Biron and van Veldhoven 2012), more capable of managing stressors (Gloster et al. 2017), and more committed to their values and actions (McCracken 2013).

A flexibility perspective on ability EI has important implications. One implication is that the attitudinal and emotional reactions of individuals with high EI may be more variable, particularly when that variability involves stimulus-appropriate responding (Hardy and Segerstrom 2017). In many cases, this variability would be linked to higher levels of distress or dissatisfaction, particularly when circumstances warrant such feelings (Matthews et al. 2006). Stated in other terms, the suggestion that high EI should support well-being (Goleman 1995) appears overly simplistic. It may support well-being under some circumstances but undermine well-being in others (Engelberg and Sjöberg 2004). Similarly, the suggestion that high EI should be linked to positive relationship behaviors (Goleman 1995) also appears simplistic. Under certain circumstances (e.g., being mistreated by one’s partner), high ability individuals would likely criticize and confront their partners (Overall and McNulty 2017). Finally, the suggestion that high EI should support better performance (e.g., at work: Goleman 1995) may need to be qualified as well. If the workplace is a threatening or stressful place, high EI individuals may—because they are more affectively attuned—disengage and underperform. In all such cases, we agree with Ybarra et al. (2014), who suggest that ability EI is likely to function in dynamic rather than invariant terms. If so, it is crucial to understand momentary situational factors in making predictions about how ability EI operates (Moeller et al. 2014).

Related to these arguments, Fiori and Ortony (2021) contend that high ability individuals may be “hypersensitive” to affective information or valence-based cues. We would suggest, however, that the distinction between appropriate sensitivity and hypersensitivity is not an easy one to make and that being attuned to affective information has demonstrable benefits according to the affect and decision-making literature (Bechara 2004; Clore et al. 2001; Pham et al. 2001). For example, if the circumstances of one’s job are either problematic or non-rewarding, it may make sense to seek other employment. Participants with higher EI levels appear to be more certain of whether they like or dislike their jobs (Irvin et al. 2023; Robinson et al. 2023) and this degree of evaluative certainty should promote appropriate behavior, such as finding another job when one’s current job is dissatisfying.

Consistent with the analysis of Fiori (2009), we see the need for further process-oriented research, and we highlight some directions that, on the basis of the present arguments and data, would seem to have merit. Chronometric paradigms should be used to determine whether participants with high EI have an easier time evaluating stimuli (Jarvis and Petty 1996) and/or are more sensitive to evaluative meaning in affective priming paradigms (Hermans et al. 2001). Sensitivity to affect is also thought to play an important role in performance monitoring and cognitive control (Teper et al. 2013) and paradigms of this type may offer valuable insights. For example, Wilkowski and Robinson (2008) found that participants with psychopathic traits were less likely to slow down following their errors in a choice reaction time task and this diminished sensitivity to error was associated with poorer performance. By contrast, we would suggest that participant with high EI would be more likely to notice and react to their errors, which should facilitate several forms of cognitive control. Performance monitoring can also be examined using EEG-related techniques, which may establish that individuals with higher EI are more “clued in” to cognitive conflicts or mistakes (Compton et al. 2008).

Affect is thought to play an important role in decision-making (Peters et al. 2006) and a number of decision-making paradigms suggest themselves. As an example, Caruso and Shafir (2006) asked participants whether they would prefer to watch a silly comedic movie or an intense, but acclaimed drama. Bringing attention to mood, which was achieved by asking participants about their mood before making a choice, was linked to higher preferences for the silly comedy, which would presumably be more enjoyable. If participants higher in EI are more sensitive to their affective states, they may be more likely to make choices that favor enjoyable experiences. Other paradigms of this type were developed by Robinson et al. (forthcoming a), who asked participants (for example) how willing they were to re-view affective images. Participants who attend to their emotions more habitually were less willing to re-view negative images and more willing to re-view positive images, in essence demonstrating greater guidance by the pleasure principle—i.e., approaching that which will elicit positive experiences and avoiding that which will elicit negative experiences (Elliot 2006). We are fairly certain that phenomena of this type would be more pronounced at higher levels of ability EI, but the relevant experiments have not been performed.

Irvin et al. (2023) found that affect–behavior and cognition–behavior relationships were stronger among participants with higher ability EI levels, but the paradigm was admittedly simplistic. We, therefore, need to know more about relationships of this type, preferably centering on consequential attitudes (such as toward health behaviors or political candidates) and their ability to predict later behaviors (Conner et al. forthcoming). We suspect that the evaluations and attitudes of high EI participants would predict subsequent decision-making and behavior to a greater extent, but the relevant studies have not been performed. We also need more research of the type conducted by Moeller et al. (2014), who found that relationships between daily negative affect levels and social behavior were stronger at higher levels of ability EI. Generally speaking, attitude–behavior and affect–behavior relationships are likely to vary positively with ability EI levels and we encourage further research on this affect–behavior interface.

The present analysis would seem to relate to the emotion perception branch of EI to a greater extent than the management branch and we certainly believe that the evaluative expertise angle that we have pursued is not sufficient in covering all of the skills assessed by ability EI tests. On this point, considerable theorizing and some sources of data have suggested that it may be healthier to experience emotions of a given valence in a more “granular” or differentiated manner (Smidt and Suvak 2015). Interestingly enough, valence focus, which we emphasize in the present paper, tends to be associated with lower rather than higher levels of emotion differentiation (Barrett 1998) and ability EI too may be linked to lower levels of differentiation (MacCann et al. 2020a). Such findings provide some support for emphasizing valence rather than discrete emotional states, as we have shown in this paper. Nonetheless, we reiterate the point that our analysis is relatively silent concerning a variety of abilities that fall within the emotional intelligence domain.

## 7. Conclusions

Both Fazio (2000) and Lewin (1935) highlighted the importance of affect to self-regulation and behavior. By affixing affective tags to objects in the environment, the person is spared from lengthy deliberation and has guidance concerning the choices that they should make (Fazio and Powell 1997). We suggest that many of these processes are likely to vary positively with ability EI, which can be profitably viewed in terms of individual differences in evaluative certainty and/or expertise

## Data Availability

Not applicable.
